# Case in Which an Attempt Was Made to Extirpate Ovarian Tumors

**Published:** 1826-08

**Authors:** 


### OVARIAN TUMOR.

### *Case in which an Attempt was made to Extirpate Ovarian Tumors.* By Dr. Granville.

Dr. Granville proceeded, on the 1st of last month, to perform an operation, with the intention of removing several ovarian tumors from a woman, formerly a patient at the Westminster General Dispensary, in the presence of Messrs. Brodie, Keate, Earle, &c. The two former gentlemen had, some time before, examined the woman in consultation with Dr. Granville, and inquired into the history and nature of her complaint. Although this case was not deemed by them a very eligible one for such an operation, it was nevertheless attempted, from a conviction that the patient's life, under the pressure of the disease, could not be saved,—nor, indeed, prolonged beyond one or two more years : whereas, if the operation succeeded, her existence might be extended to a much longer period. The patient, moreover, appeared so very desirous of a trial being made to operate upon her, and exhibited, in various respects, so many promising concurring circumstances in her constitution towards the success of the operation,—particularly a passive temperament, and an almost total absence of irritability,—that all the gentlemen present agreed in thinking Dr. Granville fully warranted in his endeavours thus to save the poor woman from the effects of an otherwise incurable malady.

The patient was prepared by a fortnight's absolute quiet and low diet, with occasional aperient medicines. She was directed to take a mixture containing hydrocyanic acid for two or three days previous to the operation; and a day was selected for performing it, when the thermometer stood at only seventy-five degrees.

Dr. Granville proceeded to make an incision through the integument, two inches above the umbilicus, in a line between it and the inner margin of the rectus muscle of the left side, which incision he extended as low down as the superpubian region, to seven inches and a half in length; carefully dissecting the various parts, until he came to the peritoneal lining of the abdomen. This he divided at the upper part of the external incision, and continued the division of that membrane downward for a space of between three and four inches; when the intestines, with part of the omentum, in rather a vascular condition, presented themselves, along with the margins of two of the tumors, right and left of the wound. It had been agreed, anteriorly to the operation, that if, on examination, after a sufficient incision, numerous and strong adhesions were detected, the separation of which would materially increase the risk of the patient, all further proceedings should be suspended. Dr. Granville, therefore, passed his hand into the abdominal cavity, and ascertained that, although the tumors (of which there appeared to be several) felt loose when examined externally, they were, in fact, adhering in various directions by strong bands. This was particularly the case with regard to the tumor which ascended on the right side towards the liver, and on the left to a large cyst which had contained ten pints of a highly albuminous fluid, that had been removed some time before by tapping, and which was found to adhere, throughout the best part of its anterior and lateral surface, to the parietes of the abdomen, and to some of the convolutions of the intestines. Both Mr. Brodie and Mr. Keate having satisfied themselves that such was the state of the parts, it was determined to go no further, and to close the wound. This Dr. Granville did by means of hare-lip pins, instead of stitches, placed at short distances; and we understand that the operator attributes, in a great measure, the rapidity with which the wound healed in the course of a few days, leaving a neat and firm cicatrix, with scarcely any point of suppuration, to this mode of dressing it.

The patient, who bore the incisions with the greatest fortitude, and who assured her physician that the pain was nothing compared with that of labour, (thereby furnishing us with a measure of the suffering experienced on occasions of this nature,) never felt a moment's uneasiness after the first night, which was rather restless. No symptom of inflammation or fever occurred; the spirits continued good; and the pulse never varied more than from eighty to eighty-two in a minute. She left her bed at the end of the week. During the incisions scarcely a table-spoonful of blood was lost.

As far as it goes, this case adds another to the many instances on record of the impunity with which the cavity of the abdomen may be laid open, and is an encouraging step towards attempting, under proper circumstances, and after mature deliberation, an operation, which Mr. Lizars, of Edinburgh, has the merit of having revived on rational grounds. Dr. Granville intends to publish this, along with some other cases, in a work on diseases of the ovary, which he has been for some time preparing.

